# College of Physicians—Session of December 7th, 1841

**Published:** 1842-03-19

**Authors:** 


### PROCEEDINGS OF SOCIETIES.

*College of Physicians—Session of December 7th,* 1841. *Continued.*

After the discussion of the paper of Dr. Parrish, Dr. Ashmead communicated a case of death from over distension of the bowels, by flatus, in which the pressure of the intestines on the great vessels and the diaphragm appear to have caused death, by embarrassing the process and arresting the mechanical operation of respiration. The case was one of inflammation of the peritoneal coat of the intestines, complicated with a partially strangulated hernia, which seemingly may have been the immediate cause of the inflammation; but the hernia was reduced without any difficulty. We have room only for a condensed view of the account of the case, and the comments of other members upon it.

"W. Wyre, aged 40 years, of large robust frame and in full health, was attacked with violent abdominal pain, vomiting and constipation, about 3 o'clock, A. M., on the 27th of August last. At 3½ P. M., I saw him; he had then feeble and frequent pulse, shrivelled skin, covered with cold, clammy sweat; hands and face blue, as in the last stage of Asiatic Cholera; respiration short and hurried; abdomen enormously inflated with gas, tense as a drum, and rising high above the level of the sternum. He was suffering under great distress and uneasiness, but had no acute pain. His intellect was perfectly clear. I found on inquiry that the patient had arrived here on the 26th, from England, after a long voyage, during the few last days of which he had been on short allowance. The night previously to his attack, he had eaten voraciously of watermellon, and drank freely of small-beer and cider just before retiring to bed."

Dr. A. suspected, discovered, and reduced a strangulated hernia. It was large and scrotal, on the left side. Repeated teribinthinate injections were given, and the attempt was unsuccessfully made to evacuate the flatus by means of the tube. The attempt to rise in bed nearly produced immediate death, and this result appears to have followed a change of position, throwing the weight of the body on the abdomen.

"During his last moments, I proposed tapping the cæcum, an operation which may be done without penetrating the peritoneum ; to this the patient consented, but unfortunately no instrument, not even a pen-knife, could be procured until too late. The whole time I was with him was not more than half an hour, and every thing had to be done by myself; could I have had fifteen minutes longer, the life of the patient might have been saved."

The abdominal appearances, (the only morbid ones) as observed on examination, were as follows:

"Peritoneum of intestines, exhibited slight injection over the stomach, duodenum, and upper half of the jejunum ; over the lower portion of the jejunum, and the ilium, the injection was much higher, approaching to a dark colour, and over the whole of the large intestines, the colour was so dark that it might have been mistaken for gangrene, had there been any odour or softening of the structure, which, however, was not apparent. A small portion of coagulable lymph was observed on the portion of the bowels which had been strangulated. Omentum natural, and pushed far up in the hypochondriac region—stomach, liver, spleen and kidnies, all healthy ; bladder contracted. The position of the diaphragm was particularly remarked. The highest point of its *peritoneal surface* (ascertained by thrusting an iron stile, through the chest, perpendicular to the spine,) after the removal of the bowels, was, on the right side *three inches above the nipple*, or half way between the nipple and the lower edge of the clavicle ; on the left side *one inch above the nipple*—bowels greatly distended, their mucous membrane throughout of a healthy texture—the duodenum and upper half of the jejunum empty, and compressed together—the lower half of the jejunum and the ilium, greatly distended with gas, and loaded with yellowish fluid fæces, and small pieces of undigested vegetable substance of the size of beans—the distension increased towards the cæcum. On the anterior surface of the ascending colon, two or three inches above the ilio-cæcal valve, its peritoneal coat was lacerated, making a tear two or three inches long, and about one and a half inches wide. The longitudinal band of muscular fibres at this point was also torn across and retracted, so as to obliterate the pouches which exist at this place. So great was the distension of the cæcum, that on cutting off the ilium two inches above it, a violent gush of fluid fæces and gas took place, attended with a loud noise ; the contents of the bowels being propelled to the distance of at least four feet from the body. The ascending, transverse, and descending colon were also enormously distended, the latter passing very high up, into the left hypochondriac region. The distension gradually lessened from the transverse colon downwards. The sigmoid flexure with its meso-colon, were highly injected and ecchymosed, the dark colour terminating above and below in abrupt lines, showing the exact extent of the stricture. The part which had been strictured was attached to the internal abdominal ring by elongated old adhesions. The lower portion of the sigmoid flexure, and the rectum were perfectly natural."

Dr. A. stated that an instrument transfixing the abdominal parieties "one inch above and one inch to the left of the anterior superior spinous process of the ilium" penetrated the cæcum about an inch outside the reflexion of the peritoneum from the intestine to the abdominal walls.

During the discussion following this case, Dr. Meigs warmly advocated the employment of the tube in evacuating flatus, especially in puerperal peritonitis. Considering these cases to be marked by paralysis of the muscular coat in general, from over distension and obstruction or spasm at the sigmoid flexure, he considers it indispensable that the tube should be made to enter the descending colon, in order to give certain relief. In addition to other cases, Dr. Meigs narrated one of colica pictonum in which life was preserved by this application of the tube.

After the close of this discussion,

"Dr. Meigs stated, that he had in his hand yesterday a placenta of ordinary size and structure of a child born at full period; attached to the edge of this placenta was another much smaller, but of an indurated structure like a gland. To the latter was attached an umbilical chord, and the two inner membranes inclosing a fœtus of about two months and a half, shrivelled and looking like a mummy. He learned from the medical friend who brought him the specimen, that the woman from whom it had been discharged had given birth to a still-born child at full term, with the placenta, &c. attached as usual, but with it this peculiar structure was thrown off. The female had been subject for several months prior to her accouchement to attacks of uterine hemorrhage. Dr. Meigs considered it a case of twins, in which one had died at two and a half months, the other continuing to live to full term. There was a death of one fœtus without abortion. He had seen a case some years since in which a fœtus of four and a half months had followed the birth of a child at full term. But such cases were very rare."

